# Intelligence Algorithms for Protein Classification by Mass Spectrometry

**DOI:** 10.1155/2018/2862458

**Published:** 2018-11-11

**Authors:** Zichuan Fan, Fanchen Kong, Yang Zhou, Yiqing Chen, Yalan Dai

**Affiliations:** School of Computer and Information Science, Southwest University, Chongqing 400715, China

## Abstract

Mass spectrometry (MS) is an important technique in protein research. Effective classification methods by MS data could contribute to early and less-invasive diagnosis and also facilitate developments in the bioinformatics field. As MS data is featured by high dimension, appropriate methods which can effectively deal with the large amount of MS data have been widely studied. In this paper, the applications of methods based on intelligence algorithms have been investigated. Firstly, classification and biomarker analysis methods using typical machine learning approaches have been discussed. Then those are followed by the Ensemble strategy algorithms. Clearly, simple and basic machine learning algorithms hardly addressed the various needs of protein MS classification. Preprocessing algorithms have been also studied, as these methods are useful for feature selection or feature extraction to improve classification performance. Protein MS data growing with data volume becomes complicated and large; improvements in classification methods in terms of classifier selection and combinations of different algorithms and preprocessing algorithms are more emphasized in further work.

## 1. Introduction

Mass spectrometry (MS) is a technique used in the study of protein. MS holds great promise for biomarker identification and cancer classification. It is necessary to employ fast and effective methods, which are greatly helpful in selecting the information that we need for the classification of MS data [1–3]. Since the amount of MS data has been dramatically growing, appropriate techniques which can effectively facilitate protein classification are urgently required. Traditional molecular and statistical techniques have failed to meet the demand of rapid and accurate classification for large amount of the mass spectrometry data [4].

Thus, protein MS classification is not only a single issue in the field of biology, but also a collaborative project staffed by researchers in various subjects and areas, such as statistics and computer science.

A number of approaches and tools based on intelligence algorithms have been developed. On the basis of the survey of current work, this paper mainly investigates several representative classification and biomarker analysis methods, including typical machine learning approaches like logistic regression, KNN algorithm, support vector machines (SVM), decision tree algorithm, and neural networks algorithm. The ensemble strategy algorithms have been discussed. Then typical preprocessing algorithms such as wavelet and genetic algorithm have been studied. It is well known that the preprocessing such as feature extraction and feature selection is a key step for protein classification. This topic which has been investigated by many studies is not the focus of this paper.

In this paper, MS-based data is introduced firstly. Then data mining classifications including representative machine learning approaches with their features and database are discussed. The ensemble strategy algorithms and some typical preprocessing algorithms also have been studied. Discussion and conclusion on the studied algorithms have been conducted in the next section.

## 2. MS-Based Data

Database plays an important role in amino acid and protein research. Several public MS-data repositories have been widely used. The PRoteomics IDEntifications (PRIDE) database [5] is one of the world-leading data repositories of mass spectrometry (MS-) based proteomics data. The Proteomics Standards Initiative (PSI) data standard formats are supported by PRIDE. PeptideAtlas [6] is another prominent MS-database. Both PRIDE and PeptideAtlas are focused on the tandem MS (MS/MS) data storage and dissemination. PASSEL [7] has focused on the SRM data. There are other MS-based repositories [8–12] in the research of proteomics. The dimensions of MS-based data are normally much larger than the number of samples. Hence, it posed a challenge on classification algorithms for protein classification.

## 3. Methods

### 3.1. Representative Classification Based on Machine Learning Algorithms

#### 3.1.1. Logistic Regression

In statistics, logistic regression is a regression model where the dependent variable (DV) is categorical. In 2004, to solve the problem that most classification algorithm for MS could not give any precise prediction of peak intensity patterns, Han Liu [13] proposed a model based on extended regression models. Robustness and efficiency were achieved by proving the coefficient vector of regression problem computed by SVD decomposition of original data. However, this approach has the problem of which the parameters have no biological meaning. In 2009, Chen Shao [14] proposed a new measurement called Oseore. It was developed by logistic regression based on a training data set produced from 18 known proteins' mixture. Oseore directly estimates the probability of a correct peptide assignment for each MS/MS spectrum. In 2017, Bart J. A. Mertens [15] presented an adaption of the logistic regression model for the evaluation of MS data in proteomics case-control studies. A fully Bayesian approach was implemented to estimate the parameters of the Gaussian basis functions which were linearly combined as considered. The calculation speed and consistency of convergence were guaranteed even though the initial value is far from the optimal solution.

#### 3.1.2. KNN Algorithm

In 2003, by comparing the performance of k-nearest neighbor classifier with some other statistical methods, including linear discriminant analysis, quadratic discriminant analysis bagging and boosting classification trees, support vector machine (SVM), and random forest (RF), Baolin Wu [35] found that the k-nearest neighbors (KNN) did not outperform in the analysis of the data set which consists of MS spectra that extends from 800 to 3500 Da, and that was obtained by serum samples from 47 patients with ovarian cancer and 44 normal patients. Then, Martin Wagner [36] improved KNN by using Mahalanobis distance to classify lung cancer and found the KNN can yield a result with lower misclassification rate compared with linear discrimination methods. In 2004, by combining the KNN with the genetic algorithm (GA), Leping Li [37] proposed a new method called GA/KNN and applied it to the classification of ovarian cancer data successfully with accuracy of 96%.

In 2015, Scott Powers [38] applied the lasso regression with KNN to a mass-spectrometric image data to detect the gastric cancer tissue with misclassification rate of 2.97%. They also came up with an idea called “customized training”: for each subset of the test data, the customized training could choose a subset that is close to this subset by clustering algorithms. In 2016, Destiny EO Anyaiwe [16] proposed the modification of Euclidean distance formula based on KNN, diversely answering the strong requirement of satisfactorily tackling spectrometer generated data. Without collaborating with clinical records, a platform designed by Destiny achieved immediate classification and indication of spectrometer data predisposed to AD with accuracy of 75%.

In 2018, Runmin Wei [39] comprehensively compared several imputation methods (including KNN) for different types of missing values using four metabolomics data sets, in order to handle the missing values which exist widely in mass spectrometry-based metabolomics data.

#### 3.1.3. Support Vector Machines

In 2005, SVM was applied by John S. Yu [29] on the ovarian cancer MS after a four-step data preprocessing. After 1000 independent k-fold cross-validations, it achieved average sensitivity of 97.38% and average specificity of 93.30%, respectively. In 2008, Michele Ceccarelli [40] tested the performance of SVM, which used radial basis functions as kernel functions on MS data from a study on female ovarian cancer patients and controls. As a result, with the Gaussian function *σ*=0.1, a size of the window that equals 3, using 8 components and having the RBF kernel functions *ν*=3, got the best mean correct classification rate, 97%. In 2008, based on the MS data provided by the organizers of the “Classification Competition on Clinical Mass Spectrometry Proteomic Diagnosis Data,” Dirk Valkenborg [41] tested the performance of 56 different methods obtained by combining eight feature selecting methods and seven classification methods. The best results with sensitivity of 0.87 and specificity of 0.82 were achieved by using linear kernel.

In 2009, by first preprocessing MS data with Kolmogorov-Smirnov test and under the restriction of coefficient of variation and wavelet analysis, Michele Ceccarelli [17] applied SVM to detect ovarian cancer. The new method was tested on the same data set and got average accuracy of 0.9818 and standard deviation of 4.314×10^−5^. In 2012, Reiner Schaumann [42] compared the performance of visual inspection, statistical similarity analysis, and SVM on the mass spectra data, which were from 76 clinical isolates of Enterobacteriaceae and P. aeruginosa. The experiments show that SVM could achieve a correct classification rate of up to 70%. However, it is worth noting that SVM-based methods may be not yet reliable enough for routine diagnostics.

In 2016, Cheryl A. Mather [30] developed SVM running with the kernlab program in R to detect Vancomycin-Intermediate Staphylococcus aureus. By testing 21 VISA, 21 hVISA, and 38 VSSA by Matrix-Assisted Laser Desorption Ionization-Time of Flight (MALDI-TOF) MS, the SVM-based model achieved a correct classification of 100% of the VISA isolates and 97% of the VSSA isolates, for an overall classification accuracy of 98%. By adding hVISA to the model, the model resulted in hVISA identification of 76%, VISA identification of 100%, and VSSA identification of 89%, for an overall classification accuracy of 89%. Cheryl A. Mather also pointed out that the new method may increase the time and costs greatly.

Zhiwei Zhou [43] applied support vector regression based on 14 molecular descriptors to predict the collision cross-section (CCS) values for metabolites, which achieved high prediction precision with median relative error about 3%. Based on this method, they also generated predicted CCS values of metabolites database.

Shotaro Kumozaki [44] presented a SVM based approach, called Structured SVM, to de novo sequence of glycans from tandem mass spectrometry. Structured SVM allows training for general structured output labels. They applied structured SVM to optimize the scoring function of the glycan structure, which enabled them to perform accurate de novo sequencing of glycans.

#### 3.1.4. Decision Tree

In 2002, Baoling Adam [18] proposed a new high-throughput proteomic classification system by developing a decision tree classification algorithm that is based on nine-protein mass pattern. The result of the experiment in which some blood samples from both prostate cancer antigen (PCA) and healthy man cohort were used to verify the validity of the new system shows that 96% of the samples can be correctly classified. In 2003, Markey [45] applied CART (Classification and Regression Tree) model to classify 41 clinical specimens based on 26 variables with accuracy of 90%. Due to the limitation of the size of the data, cautions should be paid when interpreting the preliminary results. In 2012, based on advanced decision tree logic, Derek J. Bailey [46] developed a sequence identification algorithm (inSeq) which achieved real-time prediction with an average time of 16 ms. Furthermore, using inSeq, the precision and accuracy were improved greatly. In 2013, Thomas Fannes [47] introduced cleavage prediction with decision trees (CP-DT) to identify mass spectrometry-based peptides data. CP-DT could greatly reduce the search space and significantly outperform Keil rules. In 2018, Hsin Yao Wang [48] proposed a strategy of building templates of specific types. It was used to facilitate generating predictive models of methicillin-resistant Staphylococcus aureus strain by decision tree algorithm. Predictive accuracy of all generated models was more than 0.83.

*(1) Classification Tree. *In 2003, Hong Zhu [19] presented a new tree-based disease classification method to discriminate protein by mass spectrometry. By projecting the data onto the basis by wavelet transformation and then constructing feature space by feature selection, the recursive classification tree algorithm could partition the feature space and produce smaller and smaller subsamples which are relatively homogeneous. In 2011, by combining MALDI-TOF MS with WCX magnetic beads, Chun Deng [20] constructed a classification tree model based on Leo Breiman [49] by 7626, 8561, and 8608 m/z. And the new model was applied subsequently to discriminate patients with pulmonary tuberculosis (TB) from patients with non-TB with a sensitivity of 98.3% and a specificity of 84.4%. In 2014, Yun Xu [50] put forward the classification decision tree of acute leukemia spectra which was divided into five groups (CON, APL, AML-Gra, AML-Mon, and ALL). By scoring in the maximum tree with root node M3667, they found that the proteomic-based classification of samples was consistent with the MIC-based classification for each case except for the 15th sample (M4eo) which verified the accuracy of classification results produced by the new classification decision tree.

In 2002, two boost-based classifiers were developed by YinSheng Qu [51] to separate the PCA from the noncancerous samples. The AdaBoost classifier can achieve 100% sensitivity and specificity, while the Boosted Decision Stump Feature Selection classifier, a combination of 21 base classifiers, can achieve a sensitivity (86.7-94.6%) and specificity (90.7-97.1%). In 2005, on the basis of the NIH and FDA Clinical, Ilya Levner [21] presented a nearest centroid classifier used for the analysis of MS spectra. Finally, 97.34% sensitivity and 96.99% specificity were attained on 99.92% of the test data, when 75/25 train/test split was used on the OCWCX2a data set.

*(2) Random Forest. *In 2003, Baolin Wu [35] tested the performance for a classification of ovarian cancer using mass spectrometry data by different classifiers, including LDA, QDA, KNN, bagging and boosting classification trees, SVM, and random forest (RF). The accuracy of the classification result was improved to approximately 92% when RF was applied on both feature selection and classification. However, given that the current sample size is relatively small (47 patients with ovarian cancer and 44 normal patients), it is needed to find a more robust approach. In 2004, Grant Izmirlian [52] described how to successfully apply the RF algorithm in the research of proteomics profiling. Assuming the RF with 1000 trees on simulated data which consisted of a sample of 100 spectra with each spectrum containing 138 peaks, the median (and 5th, 95th) percentiles corresponding to the error rate, sensitivity, and specificity were 32% (24.9%,40%), 76.9% (64.4%,87.1%), and 63.8% (54.2%, 73.4%), respectively.

In 2004, RF was implemented by Glen A. Satten [53] after standardization step and denoising step to classify mass spectra data of whole-organism bacterial specimens. In both test sets, by using intensities at the 3075 m/z ratios identified in the training set, all bacterial spectra can be correctly classified through this algorithm. In 2008, Jennifer H. Barrett [54] implemented RF to classify the proteomic profile which is obtained by mass spectra from 76 breast cancer patients and 77 controls. Based on peaks detected from the profiles, the RF could classify these samples into target classes with a sensitivity of 81.6% and a specificity of 85.7%. In 2010, Marc Kirchner [23] presented the RF in the phosphorylation data set. As a result, the observed real-world performances are within two standard deviations of the theoretical values. Marc Kirchner presented a nonlinear RF classification combined with a discrete mapping approach, and the observed real-world performances were within two standard deviations of theoretical values. In 2014, Suh-Yuen Liang [55] implemented RF to classify N-glycopeptides using mass spectral features form ion trap-based LC-MS2 data. By applying different sampling strategies, training sample size, and feature set, the optimal true-positive rate, precision, and false-positive rate on 577 high-confident spectral data pieces is 73%, 88%, and 10%, respectively. In 2016, Camila Maione [56] presented a model to classify the geographical original of 31 white rice samples of Oriza Sativa variety by inductively coupled plasma mass spectrometry. By applying SVM and RF, they achieved accuracy of 93.66% and 93.83%, respectively. In 2017, Dong Kyu Lim [57] detected adulterated admixtures of white rice from Korea and China by combined mass spectrometry with various machine learning algorithms, including RF, SVM with radial basis function kernel, C5.0, model average neural network, and k-nearest neighbor. In their experiment, RF and SVM with fine-tuned parameters achieved superior performance in discrimination between original sample from Korea and blended samples with accuracy generally above 95%. Albert Y. Xue [58] presented a machine-learning based method to address the problem of the large nonquantitative relationships between the peptide's amino acid sequence and peptide signal-to-noise ratios (SNR). Random forest was applied to predict the peptide's SNR by its amino acid sequence with Q-squared value of 0.59, which confirmed that the amino acid sequence could be used to predict SNR values of peptide. Mikhail Kolmogorov [59] explored an approach based on RF regression for theoretical nanospectra generation. Each element of a feature vector is a pair of volume and hydrophilicity of the corresponding amino acid quadromer. The volumes of all 20 amino acids could be used as features, because the RF model was more robust to outliers and less overfitting.

#### 3.1.5. Neural Networks Algorithm

In 2003, by using Naive Bayes with a multilayer perceptron, Melanie Hilario [25] addressed the supervised learning problem of the analysis of 24 diseased and 17 healthy specimens based on protein mass spectra. With this method, results with high predictive accuracy (1–2 off-sample misclassifications) can be produced, and greater reliability can be achieved over varying experimental conditions.

In 2008, Villmann [26] developed two classification algorithms, called the Supervised Neural Gas (SRNG) and the fuzzy labelled SOM (FLSOM) based on neural classifiers in the analysis of mass-spectrometric data. To validate the effectiveness of new methods, they have compared the performance of six methods, including a linear SVM, SVM with a radial basis function kernel, SVM with a Tanimoto-distance-kernel, LDA, SRNG, and FLSOM. The classification accuracy of these six methods was 61.5%, 34.9%, <10%, 96.3%, 97.8%, and 73.4% separately when they were applied on the listeria data set and were 59.8%, 62.8%, 42.7%, 84.2%, 80.4%, and 72.4%, respectively, when they were used on the breast cancer tissue data set. So it can be found that compared with the other four methods, FLSOM can provide comparable classification accuracy with detailed class similarity description, while SNG can achieve the best accuracy. In 2017, Chunwei Ma [27] proposed convolutional neural networks for model training. While evaluating on the test data, Chunwei Ma got the AUC of 0.96 and 0.92. In consideration of the large-scale, sparse nature of mass spectra data, Ma reduced the dimensionality using compressed sensing and validated its feasibility by robust signal reconstruction. In 2017, Krishnan [60] attacked the problem of training with reduced wall-time via a novel stochastic optimization algorithm that uses (limited) second-order information without explicit approximations of Hessian matrices or even Hessian-vector products. This large batch, stochastic optimization algorithm is faster than widely used algorithms for fixed amounts of computation and also scales up substantially better as more computational resources become available. In 2018, Samira Beyramysoltan [61] proposed an artificial neural networks method (ANNs) based on the Kohonen SOM approach for processing of DART-HRMS data in order to enable rapid discrimination and identification of fly species even for the immature life stages.

Table 1 summarizes several methods of representative data mining classification and gives their characteristics and examples, including logistic regression, KNN algorithm, support vector machines, decision tree algorithm, classification tree, random forest, and neural networks algorithm. It can be seen that typical data mining techniques are mostly based on sample databases and basic algorithms.

### 3.2. Biomarker Analysis Algorithms

Several methods of representative data mining classification applied in biomarker analysis are discussed here, including SVM, decision tree and neural networks algorithm.

In 2005, Habtom W. Ressom [62] proposed a biomarker selection algorithm for liver cancer classification through high resolution SELDI-quadrupole-TOF data. The PSO-SVM algorithm was applied to compute the optimal m/z windows from 357 sera samples. 7-9 windows were selected as biomarkers that achieved 91% sensitivity and 92% specificity. In 2006, based on SVM, Xuegong Zhang [63] proposed a new method R-SVM. R-SVM was adopting a recursive strategy which is similar to SVM-RFE. The R-SVM was more robust to noise and outliers in discovering informative biomarkers and it outperformed 5%-20% over SVM-RFE in simulation experiment. In 2015, Ling Fang [64] proposed the comprehensive plasma metabolic profiling analysis method to evaluate the potential biomarkers in a primary dysmenorrhea model combined with the method of a support vector machine which optimized the selected potential biomarkers. In 2017, Guanghui Fu [65] proposed an algorithm, where the biomarker identification was based on sparse regularization variable selection in combination with subsampling, and the classification was subsequently performed by a linear SVM classifier in the selected-variable space to obtain the maximum classification accuracy.

In 2003, an effective commercially available classification Biomarker Pattern software (BPS) was developed by Antonia Vlahou [66] to discriminate ovarian cancer based on CART. The BPS achieved accuracy of 81.5% in the cross-validation analysis. From the control group of a blinded set of samples in differentiating ovarian cancer, the accuracy is 80%. In 2008, based on BPS, Keqi Han [67] constructed the classification tree on peaks at 5808, 5971, and 7779 m/z from 89 lung cancer patients and age-matched and sex-matched 68 healthy individuals; this method achieved a sensitivity of 91% and specificity of 97% on the separated group. Vincent A Fusaro [22] implemented RF classifier to identify high-responding peptides in plasma that also demonstrated the ability for verification of MS-based biomarker by RF. The high-responding peptides were selected by protein physicochemical properties. In 2018, Felipe A. dos Santos [68] proposed carrying out GC–MS metabolomic data set based on decision tree algorithm; the application of this algorithm can contribute to the development of useful strategies to help identify antimicrobial constituents of complex oils.

In 2002, Graham Ball [24] applied a multilayer perceptron artificial neural network (ANN) (Neuroshell 2) with a backpropagation algorithm to the analysis of MS data set. This method was applied to MS of tumor grade and could produce a result with an accuracy of greater than 98%. In 2003, Mark A. Rogers [69] applied neural-network to detect renal cancer or proteins that could be potentially used as biomarkers. From surface enhanced laser desorption ionization profiling of mass spectrometry data from 218 individuals' urine samples, this model achieved sensitivity and specificity values of 98.3-100% based on either presence/absence of peaks or peak intensity values from three different types of samples. In 2015, Tomasz Ligor [70] built the dedicated software implementing a multilayer neural network by using a genetic algorithm for training. His aim was to find the potential lung cancer biomarkers. Automatic peak deconvolution and identification were performed using chromatographic data processing software (AMDIS with NIST database).

Table 2 summarizes several methods of representative data mining classification applied in biomarker analysis and gives their advantages as well as disadvantages, including support vector machines, decision tree, and neural networks algorithm. It can be seen that typical data mining techniques are mostly based on sample databases and basic algorithms.

### 3.3. Ensemble Strategy Algorithms

In 2006, on the basis of the combination of different methods, including ANN, SVM, logical analysis of data (LAD), KNN, classification and regression trees (CART), and logistic regression, Bhanot [71] tried to classify PCA samples from 253 normal and 69 PCA data pieces by using peaks as initial features and training their algorithm with both raw and pattern data. As a result, overall sensitivity and specificity of this proposed method achieved by applying 10-fold cross-validation were 90.31% and 98.81%, respectively. In 2007, Assareh [72] proposed an ensemble method, where different learning algorithms such as KNN, SVM, DT, and LDA were used as basic classifiers for hybrid ensemble strategy. Different learning algorithms were applied to different samples of training data. To evaluate the performance of the new method, it was used in the same data set of Bhanot [71] and has achieved a sensitivity of 92.55% and a specificity of 96.86% when 10-fold cross-validation was also applied.

#### 3.3.1. AdaBoost

The idea behind Adaboost is that a combination of many “weak” classifiers will generate a “strong” classifier. In 2002, Yinsheng Qu [51] proposed the AdaBoost classifier to improve the specificity of the prostate-specific antigen (PSA) test for early detection of PCA. Men with total number of 386 were analyzed by surface-enhanced laser desorption/ionization (SELDI) MS. The Adaboost classifier completely separated the PCA, achieving 100% sensitivity and specificity. In 2017, in order to enhance the efficiency of phosphorylated protein identification with tandem mass spectra, Jinjin Cai [73] tried to extract the features of amino acid properties by using AdaBoost. He got a new phosphorylated sites prediction method named AproPhos. The overall results demonstrate that their method shows about 10% higher sensitivity as well as roughly 2% higher specificity than other prediction methods, and the efficiency of phosphorylated protein identification with tandem mass spectra may be increased.

#### 3.3.2. Gradient Boosting

Zhenpeng Zhou [74] employed GDBT to distinguish different genders, ethnicities, and age from latent fingerprint samples by electrospray ionization-mass spectrometry imaging. The result from 194 samples demonstrated the accuracy of 89.2%, 82.4%, and 84.3%, respectively. Besides, Xgboost was applied to clean the raw data. In 2017, Antonia Praetorius [75] applied the GDBT-based method to draw the problem of discrimination of engineered nanoparticles (ENPs) and naturally occurring nanoscale particles (NNPs) by the inductively coupled plasma time-of-flight mass spectrometer data. By GDBT, they discovered 17 critical features and achieved desired performance.

### 3.4. Preprocessing Algorithms

#### 3.4.1. Wavelet Algorithm

Wavelet algorithm could be used as denoising and feature selection for the preprocessing of protein classification [28]. In 2003, Pietro Lio [76] discussed the application of wavelet transformation in biology data analysis and summarized the potential of useful wavelets. In 2005, wavelet analysis was applied by Yu [29] as part of data preprocessing method to reduce the dimension of data. The MS data original consisted of 95 control samples and 121 cancer samples; the dimension of the original feature space is over 370 000. Discrete wavelet transformation (DWT) was applied after binning, Kolmogorov–Smirnov test, and restriction of coefficient of variation, which further compressed the dimension of data from 6757 to 3382.

In 2007, ShuoChen [77] presented wavelet-based procedures for MS data processing transforms. Stationary discrete wavelet transform (SDWT) was applied for denoising and dimension reduction for 62 healthy mice and 77 mice with tumors collected at Vanderbilt Ingram Cancer Center. After SDWT, the dimension of data was reduced from about 20000 to 1800. Then SVM with linear kernel was applied on the reduced features, which achieved 99.3056% accuracy.

In 2008, Deukwoo Kwon [78] presented a wavelet-based method for the preprocessing of mass spectrometry data to deal with heterogeneous noise. They showed that the performance of peak detection could be improved by the procedure for local wavelet thresholding of MS data.

In 2015, Dual Tree Complex Wavelet Transform using almost symmetric Hilbert pair of wavelets was proposed to denoise MS/MS data, which outperformed discrete wavelet transform (DWT) and stationary wavelet transform (SWT) [79]. In 2017, Yahya Izadmanesh [80] applied DWT to reduce the size of GC*∗*GC-TOFMS data. The DWT could capture the time property of GC×GC-TOFMS data.

#### 3.4.2. Genetic Algorithm

Genetic algorithm (GA) is an optimization procedure that repeatedly evolves a population of candidate solutions based on the natural selection law to solve an objective function. The dimension of MS-based data is normally much larger than the number of samples. Feature selection could be applied by GA. In 1996, David Broadhurst [81] applied GA as feature selection algorithm for pyrolysis mass spectrometry. GA is used to find the optimum subset of regression variables for different models, including multiple linear regression (MLR) and partial least squares (PLS). The variables were reduced from 150 to fewer than 20.

In 2002, Emanuel F Petricoin III [82] proposed a new algorithm which was developed by combining GA first described by Holland [31] and cluster analysis methods presented by Kohonen [32, 33] to detect early-stage ovarian cancer. The method was applied to select between 5 and 20 m/z peaks from 100 control samples from the National Ovarian Cancer Early Detection Program (NOCEDP) clinic at Northwestern University Hospital (Chicago, IL, USA) and 17 other control samples from anonymous women unaffected by cancer. The detection sensitivity and specificity are 95% (CI 93–100) and 95%, respectively.

In 2003, Keith A. Baggerly [83] used GA with Mahalanobis distance to classify disease spectra samples and normal spectra samples. On the basis of three peaks, including 3077, 12886, and 74263, which were selected by the preprocessing of data, this proposed method was able to achieve an overall classification accuracy of 92.6% on the data set which consisted of both raw MALDI spectra and preprocessed lists of peak locations and heights. In 2004, on the basis of GA, Neal O. Jeffries [84] developed a new method called Best GA which performed best in terms of the prediction of the omitted cases. Furthermore, the new method was tested on DS1 data set consisting of 162 ovarian cancer samples and 91 control samples, and the test set accuracy of the models with the 25th and 75th percentiles was 97% and 99%, respectively.

In 2008, Senyung Hsieh [85] tried to embed the Quick Classifier (QC), SVM, and GA into the ClinProTools software to generate models, which were used to analyse MS of bacterial isolates collected and characterized by the Clinical Pathology Laboratory of Chang Gung Memorial Hospital, Taoyuan, Taiwan. Through a series of experiments that were used to test these models constructed by all methods, they have found that optimized GA had 100% recognition capability and 99% cross-validation achievement and 100% positive predictive value.

In 2011, Elon Correa [86] proposed a genetic algorithm-Bayesian network approach for the identification of Bacillus spores and classification of Bacillus species. By combing Bayesian network and GA, the variables were significantly reduced from 150 to 22-39 depending on the subset of data. They showed that GA-BN was able to discover biomarkers for spores.

In 2017, Zhihua Li [34] proposed a built-in mathematical model of GA, SNN, and QC, to select each peptide peak. Classification models were established by using ClinPro Tools 2.1 software to determine the optimal separation hyperplane for classification. Zhihua Li identified peptide/protein differences in serum samples from SCLC patients and healthy individuals and established a serum peptide-based classification of SCLC patients with high sensitivity and specificity using MALDI-TOF-MS system.

In 2018, Ana C. O. Neves [87] presented a certain multivariate analysis based on PCA and GA with SVM, linear, and quadratic discriminant analysis. Mass spectrometry coupled with multivariate analysis was used as an untargeted lipidomic approach for classifying 76 blood plasma samples into negative for intraepithelial lesion or malignancy and squamous intraepithelial lesion.

Table 3 shows the features and samples of wavelet algorithm and genetic algorithm.

## 4. Discussion

Traditional data mining classification techniques based on protein MS data have addressed the basic needs of researchers in the field of biology. The information of protein is indexed in data sets and sorted by supervised clustering methods. Some classifications could be conveniently performed by using these algorithms. KNN algorithm is a nonparametric method for classification. An object is classified by a majority vote of its neighbors. For high-dimensional MS data, dimension reduction is usually performed prior to applying the KNN algorithm in order to avoid the effects of the curse of dimensionality. Combining KNN algorithm with Mahalanobis distance or GA, it is easy to classify multimodal sample sets which do not need to estimate parameters, but it needs a large amount of computation.

Neural networks algorithm has strong robustness and fault tolerance to noise and can fully approximate complex nonlinear relationship. Training of ANNs can potentially be time-consuming depending on the complexity of the data. Overfitting is another problem in ANNs, which requires the feature selection algorithms, like GA and wavelet algorithm. Another problem related to ANNs is that it is not always apparent how they achieved a good solution.

SVM plays an important role in data mining methods. It can improve the accuracy with its well-selected informative features. SVM can combine with other projects to preprocess the data, and it can improve generalization performance, to solve high-dimensional problems and nonlinear problems. More importantly, there is no universal solution to nonlinear problems and kernel function must be handled carefully. Linear combinations of different kernels could improve performance over the individual kernel. SVM could be considered as a first choice of classification algorithm in MS-based data since it is designed to perform well in spaces where the number of features is typically much larger than the number of samples.

The decision tree can generate understandable rules to make feasible and good results for large MS data sources in a relatively short period and it only needs to be built once, repeated use. The decision tree algorithm is a “white box” model, where the decision rule is determined after the MS-based data is given. However, there is still a problem, overfitting, in decision tree model. However, it could be solved by RF. RF uses voting mechanisms of multiple decision trees to improve the decision tree. It has a high accuracy and efficiency for a classification of high-dimensional data and can also be used as a selection of feature importance.

AdaBoost has high precision, which uses weak classifiers based on different algorithms for cascading. Compared to the bagging algorithm and RF algorithm, AdaBoost fully considers the weight of each classifier. Logistic regression is a type of regression method, which can predicate the peak intensity patterns exactly and simplify the decomposition.

The wavelet-based algorithm could be applied to the preprocess of MS data as denoising and feature selection. Denoising is an important preprocessing for classification of MS-based data. Noise signals normally from the instrumental interference, measurement, and baseline distortions. Wavelet-based denoising algorithm could handle chemical and instrumental noise. For heterogeneous noise, wavelet-based data could also achieve high performance. Wavelet algorithm was normally applied to MALDI-TOF and SELDI-TOF MS-based data. Compared with traditional PCA and LDA methods, wavelet feature selection could keep the time property, detect the localized features, and reduce the computational load of MS data.

GA search from the group, with the potential for parallelism, can compare more than one individual at the same time, and it has good robustness. It has great extensibility so that it is easy to combine with other algorithms such as SVM. GA can optimize SVM's selection of parameters and make SVM more widely used. In addition, its model has high sensitivity and experimental crossover performance.

Excluding traditional methods, there were also many other methods proposed by researchers in the last few years and could achieve high accuracy when they were applied to the analysis of MS. Ensemble strategy combines with other learning algorithms. KNN, SVM, DT, and LDA were used as basic classifiers and a hybrid ensemble strategy. Different training data samples were applied to different algorithms. Software method uses different soft wares to classify MS data such as MALDI TOF MS and MALDI BioTyper™ software. Gradient boosting produces a prediction model in the form of an ensemble of weak prediction models, typically GDBT and Xgboost. GDBT and Xgboost can be used for classification, feature selection, or data cleaning.

Additionally, deep learning has improved the state-of-the-art results in many domains, leading to the development of several systems for bioinformatics. Deep learning has the ability to automatically discover complex patterns direct from the raw data. Deep convolutional neural networks had been applied for tumor classification in IMS data [88], which indicated that deep learning method could be applied for further development in IMS data.

## 5. Conclusion

In this paper, the classification methods of protein MS data based on computational methods have been studied, including traditional machine learning algorithms, ensemble strategy algorithms, and preprocessing algorithms. As the performance of the traditional data mining algorithms relies on the resistance, uncertainty, integrity, and uniformity of data set, simple and basic methods based these machine learning algorithms hardly addressed the various needs of protein MS classification. Classification results with higher accuracy and efficiency could be achieved with the deepening of the research in the field of machine learning methods. At the same time, some new approaches, such as ensemble strategies or combinations of different kinds of intelligence algorithms, have been developed to improve the classification methods. Preprocessing algorithms such as wavelet and genetic algorithm have been widely studied, as these methods are useful for feature selection or feature extraction to improve classification performance. Since protein MS data becomes complicated and large, classifier selection, combinations of different algorithms and preprocessing algorithms are more emphasized in further work.

## Figures and Tables

**Table 1:** Typical classification algorithms and their characters and samples.

Method	Feature	Samples
Logistic Regression	can predicate the peak intensity patterns exactly and simplify a SVD decomposition [13].	Tandem mass spectrometry

KNN algorithm	by Euclidean distance or by one minus correlation. [11]	ovarian cancer MALDI-MS database
	a modification of Euclidean distance formula [16].	patients with mild cognitive impairment and patients with clinical symptoms of Alzheimer's disease [16].

Support vector machines	using 4 genes	colon cancer database
	suitable for noisy high-throughput proteomics and microarray data and outperforming in the robustness to noise	SELDI-TOF-MS
	an unsupervised feature selection phase, restriction of the coefficient of variation and wavelet analysis for classification [17].	ovarian cancer database [17].

Decision tree algorithm	a new high-throughput proteomic classification system, and developed by a nine-protein mass pattern [18]	blood samples from prostate cancers and healthy man cohort [18]

Classification tree	partitioning the learning sample into smaller and smaller subsamples to ensure the disease status within each subsample is relatively homogeneous [19].	clinical specimens [19].
	combining MALDI-TOF MS with WCX magnetic beads, and with high sensitivity (98.3%) and high specificity (84.4%) [20].	patients with pulmonary tuberculosis [20].
	boosted feature extraction coupled with the nearest centroid classifier with high accuracy [21].	OCWCX2a [21].

Random Forest	used as both feature extractors and classifier and suit for the small sample [4].	serum samples from patients with ovarian cancer [4].
	a complex proteome with a wide range of protein concentrations [22].	signature peptides [22]
	nonlinear random and combined with a discrete mapping approach [23].	phosphorylation data set [23].

Neural Networks algorithm	a multilayer perceptron ANN with a backpropagation algorithm [24].	SELDI-MS data [24].
	using Naive Bayes with a multilayer perceptron [25].	mass data set with InfoGain and Relief-F [25].
	basing on SRNG and FLSOM [26].	breast cancer listeria and tissue data set [26].
	convolutional neural networks [27].	Q-TOF and IT [27].

**Table 2:** Biomarker analysis algorithms and their advantages as well as disadvantages.

Method	Advantages	Disadvantages	Samples
Support Vector Machine	High robustness to noise and good ability to recover informative features, could work well on nonlinear problems. Stable classification rate of candidate biomarkers, high classification accuracy	Inferior in terms of the number of recovered informative genes, must according to the collaborative information of multiple genes, hard to train and hard to find kernel function	Noisy high-throughput proteomics and microarray data set Sphingosine and progesterone Metabolomics datasets

Decision Tree	easy to interpret, nonparametric method	May be stuck in local minima, overfitting data, could not be learned online	Volatile oils and S. mutans

Neural Networks algorithm	Identify masses that accurately predict tumour grade, high cross-validation on test data sensitivity rate and specificity rate	Need huge volume of samples, computational expansive to train, black box model, overfitting, hard to select meta-parameter	Astrocytoma Volatile organic compounds

**Table 3:** Traditional preprocessing algorithms for data mining classification.

Method	Feature	Samples
Wavelet algorithm	could capture localized features and keep the time property [28].	Ovarian cancer identification [29].

Genetic algorithm	discriminating VISA and VSSA straining by using the peaks that met the selection criteria described [30].	MALDI-TOF MS [30].
	combining genetic algorithm and cluster analysis methods [31–33].	NOCEDP [32, 33].
	five peptides/proteins from the training group to classify, used to select each peptide peak, and using software to determine the optimal separation planes [34].	serum samples, from SCLC patients [34].

## Data Availability

No data were used to support this study.
